# There is no ideal place, but it is best to deliver in a hospital: expectations and experiences of health facility-based childbirth in Imo State, Nigeria

**DOI:** 10.11604/pamj.2020.36.317.22728

**Published:** 2020-08-21

**Authors:** Uchenna Chinenye Gwacham-Anisiobi, Aduragbemi Banke-Thomas

**Affiliations:** 1 Health Strategy and Delivery Foundation, Owerri, Imo State, Nigeria,; 2 Department of Public Health, University of Liverpool, Liverpool, United Kingdom,; 3 Department of Health Policy, London School of Economics and Political Science, London, United Kingdom

**Keywords:** Maternal health, experience of care, maternity, childbirth, qualitative, health care provider, respectful maternity care, quality of care, Nigeria, low- and middle-income countries

## Abstract

**Introduction:**

annually, about 67,000 of the 196,000 maternal deaths in sub-Saharan Africa occur in Nigeria, second only to India. Though health facility childbirths have been linked with improved health outcomes, evidence suggests that experiences of care influence future use. This study explored the expectations and experiences of health facility childbirths for mothers in Imo State, Nigeria.

**Methods:**

this qualitative study utilised in-depth interviews with 22 purposively sampled mothers who delivered in different types (private and public) and levels (primary, secondary, tertiary) of health facilities in Imo State. Interviews were digitally recorded, transcribed verbatim and analysed following Braun and Clarke´s six-stage thematic analysis.

**Results:**

four key themes emerged from the analysis. Generally, women saw value in facility-based delivery. However, they had varying expectations for seeking care with different care providers. For those who sought care from public hospitals, the availability of “experts” was a key driver. While those who used private facilities went there because of their perceived empathy and dignity. However, while experiences of disrespect, abuse and health worker expectation for them to cooperate were reported in both public and private facilities, long waiting times, unconducive environments, and lack of privacy were experienced in public facilities.

**Conclusion:**

every woman deserves a positive experience of childbirth. To achieve this, mothers´ perceptions of different providers need to be heard. Going forward, strategies ensuring that both public and private sector providers can guarantee holistic care for every woman will be key to realising the maternal mortality target of the Sustainable Development Goal 3.

## Introduction

Daily about 540 women die in sub-Saharan Africa (SSA) from preventable pregnancy and childbirth-related causes [1]. With a maternal mortality ratio of 542 per 100,000 births [2], SSA accounts for over two-thirds (68%) of the global maternal deaths annually [3]. Nigeria contributes disproportionately to maternal deaths in SSA, accounting for 67,000 of the 196,000 maternal deaths recorded in the SSA in 2017 [2]. The 2018 demographic health survey estimates that 556 maternal deaths occur for every 100,000 live births in Nigeria [4].

Evidence suggests that quality obstetric care in the period just before, during and immediately after birth is critical, as three-quarters of maternal deaths occur in this period [5]. Health Facility-based childbirths in centres which provide emergency obstetric care (EmOC) has been associated with maternal death reductions [5]. However, the coverage for health facility-based childbirths in SSA pales at 22% [6] in comparison with the global coverage (76%) [7]. In Nigeria, only 39.4% of childbirths occur in health facilities with significant geographical disparities, which have persisted over time [8]. Imo State located in south-eastern Nigeria has the highest percentage of health facility births in the country (94.5%) [8] with a higher preference for private providers (71%) versus public providers (24%) [8]. As countries in SSA strengthen their health systems and commitment to increasing health facility childbirths, insights from regions with consistent high demands will become invaluable.

**Figure 1:**
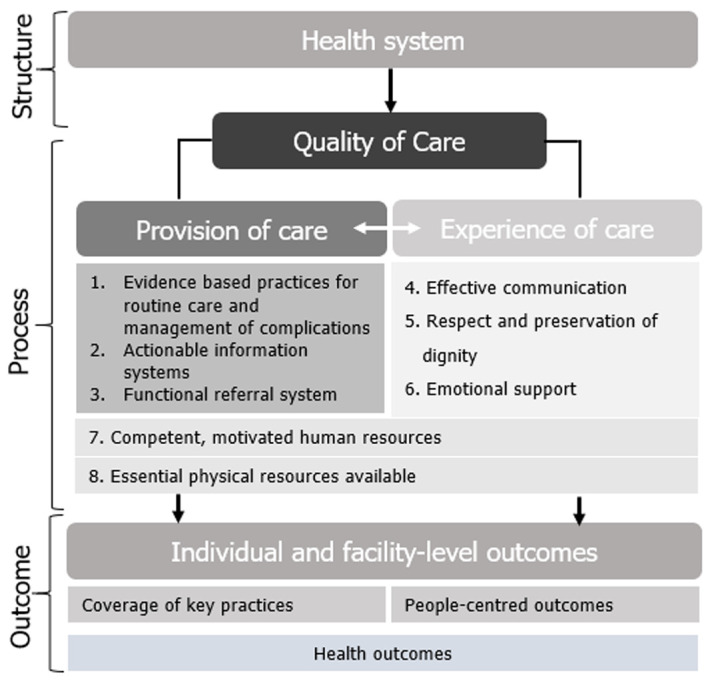
WHO framework for the quality of maternal and newborn health

Historically, assessment of maternal health indices has centred on the coverage of critical lifesaving skills (such as skilled birth attendance) and health outcomes. However, current evidence calls for measurements which focuses on what matters to care users, including the experience of care (EoC) [9,10]. Though not routinely measured, EoC has been found to bolster or hinder decisions for future access [11]. The World Health Organisation in 2016 put forward the Quality of Care (QoC) framework for maternal and newborn care in health facilities of which the EoC is a critical component in addition to the actual provision of care (Figure 1) [12]. Several studies assessing EoC during childbirth in SSA have focused on specific aspects of care, and to date, few studies have utilised the WHO framework to explore the experience of care holistically [11]. The objective of this study is to explore the expectations and experiences of health facility childbirths for women who utilised different types (private and public) and levels (primary, secondary, tertiary) of health facilities in Imo State.

## Methods

This study was conducted in Owerri, Imo State's capital, located in south-eastern Nigeria. Owerri has the highest concentration of health facilities in the State and is home to people from various socioeconomic strata [13]. With a fertility rate is 4.5 births per woman [8], the State is home to about 5.6 million people as estimated in 2017 [13]. Imo has a mixed health care delivery system at the primary and secondary levels, with key players emanating from the government, private (including faith-based) providers and development partners [13]. The researchers targeted and included women aged 18-49 years old, who had successful facility childbirths in Owerri two to twelve months before the study. Mothers below 18 years or those who had bad health outcomes (e.g. stillbirths) were excluded on ethical grounds. A maximum variation purposeful sampling was adopted to ensure that unique experiences associated with health provider types and facility levels are uncovered [14].

The participants were recruited via announcements at immunisation clinics, flyers on hospital billboards and direct verbal invitations. We scheduled interview dates (between September 2019 and October 2019) and location to suit each woman who consented to partake in the research. The women were not coerced to participate in this study, and no form of incentive (financial or in-kind) was given. The WHO Better Outcomes of Labour Difficulty (BOLD) project interview guide for health facility childbirth experience, which has been validated and utilised for several studies in Nigeria and other SSA countries was adopted for use in this research [11]. The instrument was piloted using two women meeting the study criteria to ensure its suitability, and following peer discussions, no changes were made, and these interviews were included in the study [15]. All participants consented to audio recorded sessions, and the researchers also took parallel field notes. During interviews, the researchers avoided directing the discussion and reduced social desirability bias using preliminary questions to establish rapport and also utilised a mix of direct and indirect questioning (third party referencing) as necessary [16]. Most interviews were conducted in English language; however, five interviews were partly conducted in Igbo language, which is also the mother tongue of UG. Each interview was transcribed soon after completion, employing translation and back translation for interviews conducted in Igbo language to ensure cross-cultural equivalence, preserving the participants' actual meaning [17]. Transcripts, audio recordings and field notes were used for data analysis.

After transcription, the researchers reduced the data thematically by summarising and synthesising key impressions on the transcript. The researchers utilised the Braun and Clarke six-step approach (familiarisation with data, generating codes, search for themes, defining and naming themes, and generating a report) to detect and describe implicit and explicit notions in the transcript [18]. The researchers familiarised with the data and validated transcription by listening to the audio recordings severally. The transcripts had sufficient margins which enabled the researchers to generate initial codes at this stage using an inductive open coding, without forcing data into codes identified in the literature [19]. The transcripts were analysed as proxies for the experiences, feelings and perceptions of the participants while reflecting on field interactions with each participant. A spreadsheet was used to index the generated codes and further grouped them into categories with the aid of NVivo 12 (QSR International, Memphis, TN, USA). The researchers ensured that categories were consistent within and divergent between categories [20]. Thereafter we searched for themes, tested our emerging understanding of each theme, sought alternative explanations, and our data analysis report was written [21]. Ethical approval was obtained from the research and ethics committees of Federal Medical Centre Owerri and the University of Liverpool.

## Results

**Distribution of participants**: twenty-two participants were recruited for this study. Their ages ranged from 22 to 39 years, all having a minimum of secondary education. Twenty-one of the participants were married while one is cohabiting with her partner. Interview duration ranged from 20 to 45 minutes. Table 1 shows the other sociodemographic characteristics of participants.

**Table 1:** characteristics of participants P1-P22 are the anonymised code numbers given to the participants in this study

P/No	Age	No. of facility births	No of non-facility births	Highest educational attainment	Marital Status	Health Facility used	Employment status
**P1**	30	2	0	>Secondary	Married	Private secondary	Self-employed
**P2**	35	3	0	>Secondary	Married	Public tertiary	Employed
**P3**	36	3	0	>Secondary	Married	Public tertiary	Unemployed
**P4**	27	3	0	Secondary	Married	Private secondary	Employed
**P5**	22	1	0	Secondary	Married	Public secondary	Unemployed
**P6**	39	5	0	>Secondary	Married	Public secondary	Employed
**P7**	37	4	0	>Secondary	Married	Public tertiary	Employed
**P8**	24	1	0	>Secondary	Cohabiting	Public primary	Self-employed
**P9**	26	1	0	>Secondary	Married	Public primary	Student
**P10**	35	2	0	>Secondary	Married	Public primary	Unemployed
**P11**	26	2	0	>Secondary	Married	Private secondary	Student
**P12**	27	2	0	>Secondary	Married	Private secondary	Self-employed
**P13**	34	1	0	>Secondary	Married	Private secondary	Employed
**P14**	36	4	0	>Secondary	Married	Private secondary	Self-employed
**P15**	28	2	1	Secondary	Married	Public primary	Unemployed
**P16**	25	2	0	Secondary	Married	Public primary	Petty trader
**P17**	24	2	0	Secondary	Married	Public secondary	Unemployed
**P18**	26	2	0	>Secondary	Married	Public tertiary	Petty trader
**P19**	37	3	0	>Secondary	Married	Public primary	Employed
**P20**	32	3	1	>Secondary	Married	Public tertiary	Unemployed
**P21**	29	1	0	>Secondary	Married	Private secondary	Employed
**P22**	38	3	0	>Secondary	Married	Public secondary	Employed
P1-P22 are the anonymised code numbers given to the participants in this study							

**Experience of mothers during health facility-based childbirth**: the four key emerging themes from this study are: 1) women's expectation of care during facility childbirth; 2) considerations in deciding where to seek care; 3) women's experiences of childbirth; 4) perceived expectations of health workers.

**Women's expectation of care during facility childbirth**: participants desired to have skilled health providers at the time of birth. Mothers who sought care in public referral (secondary and tertiary) facilities prioritised the availability of obstetricians. They felt they would make timely decisions on their care, and they also deemed that having many doctors and nurses working in groups will “maximise the chances for good outcomes” (Table 2, P2, public tertiary facility). However, most participants believed that positive health outcomes are hinged not only on the availability of skilled personnel and essential equipment but also on receiving the right and timely care at these facilities (Table 2, P5, public secondary facility and P14, private secondary facility). Mothers who sought care in private hospitals or public primary health centres did so based on the perceived expertise of identified care providers at these facilities. The interactions between the mothers and the health workers during the antenatal period frames the expectations of quality before childbirth and may inform the decision to continue accessing care in such facilities (Table 2, P11, private secondary facility). It appeared that many women who used private hospitals previously sought the renowned expertise at the public referral facilities either in the present or previous pregnancies. However, due to the negative experiences such as long wait times, poor provider attitudes, these mothers opted for private facilities and in most cases, one where a specialist from the referral facility also provides services (Table 2, P11, Private secondary facility and P12, private secondary facility). All participants desired friendliness, warm reception, physical and emotional support from health workers during labour. They wanted companionship from health workers, who will also teach them what to expect and reassure them. While mothers who sought care in primary centres and private facilities were almost certain they would receive the desired social support, mothers who utilised public referral facilities anticipated inadequate social interactions which they perceived as norm due to the patient volume (Table 2, P3, public tertiary facility). There was a general expectation amongst participants that they will have their family members, who they felt should provide emotional, physical, spiritual and financial support, with them in the facility (Table 2, P16, Public primary facility).

**Table 2:** illustrative quotes for themes 1 and 2

Theme 1
“You know [public facility] is where you see many doctors; you see consultants, and they work in groups” (P2, public tertiary facility).
“Once they have checked that the woman cannot push, they will not waste time they will operate on the woman immediately. The mother and baby will be alive” (P5, public secondary facility).
“You can lose your life if it takes time for the doctors to come. In the hospital I used, they had the equipment and the doctors were always around” (P14, private secondary facility).
“I came to [public facility] a few times for antenatal. The way they attended to me was shocking. The nurses were too harsh. Also, the number of hours I waited for my turn was unbelievable. I knew I couldn´t continue there. I went to a private facility” (P11, private secondary facility).
“Unlike [public facility], the Nurses in private hospitals socialise with all patients, whether you are an acquaintance or not. They give equal care to all patients” (P11, Private secondary facility).
“In [public facility], if you don´t know anybody during antenatal, things are slow for you. You can spend the whole day there on antenatal. So, I chose private that also has specialist” (P12, private secondary facility).
“But I know that some people complain that when they come, if they are crying, they [health workers] will not be that caring, but I know that is [public facility], it is straight to business. You are on your own” (P3, public tertiary facility).
“Even from the period of pregnancy, the family is very important. They will support you at home and your husband too will also pay the bill” [P16, public primary facility).
**Theme 2**
“There is no ideal place, but it is best to deliver in a hospital. Every delivery depends on God...I thought I would have delivered in the church because I was there for one month” (P9, public primary facility).
“Once my husband is in-between, and he says please I don´t want this place, straight to FMC that´s where I want you to stay and deliver” (P3, public tertiary facility).
“I think it is more about the women trying to give themselves an edge...the social status. Everyone wants to feel big here, nobody wants to look poor by going to deliver in maternity [Traditional Birth Attendants]. They also care about the safety of their lives too” (P21, Private secondary facility).
“In some cases, mothers in other hospitals which do not have the necessary facilities, are transferred to this place [public facility]. That´s why I chose here since it serves as a last resort” (P3, public tertiary facility).
“Like now I live around here, and the [primary] health centre is here. For me it makes no sense going to a hospital that is far from here, and on reaching there, you are asked to purchase a card and wait for your turn. The Nurses here are very good too” (P8, public primary facility).

**Considerations in deciding where to seek care**: the fear of bad outcomes was pervasive among participants and shaped the ideals for the right place for childbirth. All participants agree that delivery in a health facility guarantees better health outcomes for themselves and their babies. While all the women ascribed sovereignty to God, they mostly held the view that God uses health workers to accomplish his purpose of granting them safe deliveries. One participant who had a complicated pregnancy, however, esteemed deliveries in locations with *"God's presence"* as the most critical consideration (Table 2, P9, public primary facility). In discussing the perceived preference for health facility childbirth in Owerri, most participants felt it was due to women's enlightenment and desire for good outcomes. However, one participant who recently moved to Owerri believed it was more for social status statement than for safety (Table 2, P21, Private secondary facility). With many mothers paying out of pocket for medical care, participants who utilised the public facilities considered the cost implications of care. Some women who used referral facilities chose to bypass the lower levels of care as this saves them from multiple out-of-pocket spending in case of complications during childbirth. Mothers also considered the proximity and ease of access to such centres from their homes (Table 2, P3, public tertiary facility and P8, public primary facility).

**Experiences of childbirth**: all participants agreed that receiving adequate attention and support during labour was central to a positive EoC. Participants who delivered in busier public referral facilities felt they received less attention in comparison with their counterparts who delivered in privately-owned facilities. First-time mothers were also more desirous of attention and empathy from health workers in comparison with participants who have had two or more childbirths (Table 3, P3, public tertiary facility). The participants felt better when health workers attending to them were friendly, engaged them in conversations which distracted them from their immense pain and when they adopted a *"motherly"* role of patiently guiding them through the course of labour and delivery. Though most mothers using private facilities anticipated and enjoyed this level of support, it was a pleasant surprise for one mother using a public referral facility (Table 3, P13, private secondary facility and P7, public tertiary facility). Women who delivered in public hospitals argued that health workers in private facilities would be more friendly as they envisaged that the hospital management set up systems to ensure that patients are treated right. They reported that these feedback systems are either weak or deficient in public facilities (Table 3, P2, public tertiary facility and P19, public primary facility). Various forms of disrespect and abuse were reported by women who utilised care in all types and cadres of health facilities. The most common form was verbal abuse from health workers, reported most frequently by women who used public facilities. The verbal abuse received ranged from speaking in raised voices to hurling demeaning words at the care users (Table 3, P5, public secondary facility and P2 public tertiary facility). Physical abuse was also reported by mothers who used both private and public facilities. Most mothers reported receiving or witnessing slaps on the thigh while pushing, and one mother was overtly beaten with a broom. All the participants disapproved of any form of abuse, though some felt it was necessary when directed at other mothers for not cooperating with health workers. However, only mothers who used private facilities felt that their grievances were well resolved before discharge (Table 3, P6, public secondary facility and P12, private secondary facility). Though participants' interaction with health workers contributed the most to suboptimal EoC in public referral facilities, participants still argued that the overall experience is dependent on the health workers on call during confinement (Table 3, P19, public primary facility). Participants who delivered in public facilities at the onset of health workers' strike action expressed utter dissatisfaction at the abandonment in care experienced. Mothers using public referral facilities usually incur additional expenditure as they register in private facilities also in case of unprecedented strike action during childbirth (Table 3, P7, public tertiary facility). Long wait times in public referral facilities reduced access to care providers, unlike private facilities, where participants felt they had better access. Participants argued that non-availability of doctors at the public primary facilities could also lead to bad outcomes as some participants had long waits for doctors upon developing complications during childbirth. Mothers who used public referral facilities suggested that doctors working in dedicated units further complicated the wait times because women could not be attended to by doctors from another team even when they are available (Table 3, P10, public primary facility and P2, public tertiary facility). Unconducive environment more often reported in public facilities added to negative EoC, including dirty toilets, power outages, narrow beds, lack of privacy etc. In public referral facilities mothers felt that the organisation of the labour rooms and the volume of clients made it impossible to attain their desired privacy. Women who delivered in primary health centres and private facilities however felt they had privacy during confinement (Table 3, P7, public tertiary facility and P22, Private secondary facility). The mode of payment for services rendered also influenced EoC for some participants. In public facilities mothers felt they got better healthcare at affordable costs because of their health insurance cover. However, in private facilities one participant reported a reduction in the quality of care in comparison with her last confinement in the same facility, inferring her change in payment mechanism to health insurance precipitated this (Table 3, P1, private secondary facility).

**Table 3:** illustrative quotes for theme 3 and 4

Theme 3
“I think people prefer private hospital because when they do ‘ayy ooo’ [wince in pain] they have someone [health workers] that will tell them sorry, but in [public facility], it is straight to business” (P3, public tertiary facility).
“Because they are private, they attend to women well” (p13, private secondary facility).
“I have met a lot of nurses in this (public hospital), but the last one, I felt like I was with a mother...I just told her mum please be with me. She said, I am not going anywhere” (P7, public tertiary facility).
“I believe it is because it is a public hospital. I don´t think it happens in private. If all nurses were in private hospitals, I don´t think they will behave like that. They will not want to be sacked” (P2, public tertiary facility).
“In [public facility] there is no rapport with health workers. But private is looking for customers, they give the patients quick attention. Again, the medical director will not take that [poor attitude]” (P19, public primary facility)
“The nurses were saying: look at this one, what is she doing? She said she is pushing, is it not her mate that use to deliver in the bush and throw the baby away? So, I was very confused” (P5, public secondary facility).
“Instead of the nurse to carry my baby, she was busy shouting at me: why did you do this? why did you defecate on the [delivery] table? But my baby´s head was touching it too” (P2, public tertiary facility).
“Some mothers come to give birth without the hospital requirements, and this upsets health workers. They shout on them because it obstructs their work, imagine if the baby is in danger” (P6, Public Secondary facility).
“I talked to the matron about it [bad health worker attitude] too. After she spoke with the Nurse, we later became friends” (P12, private secondary facility).
“The attitude you get really depends on the individuals on duty, you can´t generalise” (P19, public primary facility).
“After delivery, I was still in the labour room, they didn´t clean me up... at a point I was now shivering. They said they have gone on strike” (P7, public tertiary facility).
“What I want is just for them to have doctor that is fully on ground there... My baby could not breathe very well when he was born. We had to wait for the doctor to come” (P10, public primary facility).
“You don´t see doctors. Even at discharge, I was waiting for my own doctor to come” (P2, public tertiary facility).
“There was no privacy. It was an open hall. Imagine I was coming in and I saw a woman delivering. Although they covered with screens, but I saw everything and that is not nice” (participant 7, public tertiary facility).
“Me I like my privacy. I told them [her family] that I cannot deliver in that FMC” (P22, Private secondary facility).
“I used insurance... when they learn that you are not paying them from your pocket, the care is reduced. The insurance is a government thing, and they may not pay them the amount they really want, the care is somehow reduced compared to the first time” (P1, private secondary facility).
**Theme 4**
“Respect and cooperation are what they require. When you respect someone, and talk to them politely, they are the ones in charge of your life. They will do their best to take care of you” (P1, private secondary facility).
“What I mean is bribery! You get better care when a mother bribes one of the nurses” (P2, public tertiary facility).
“Women should always feel confident that they [health workers] can render any assistance that they need.... But when you don´t believe in someone and request for someone else, that is not good” (P9, public primary facility).
“Health workers want me to be able to control myself, not shouting, and wasting the energy. I think it is best for me to behave normal (P3, public tertiary facility).

**Perceived expectations of health workers**: participants perceived that health workers had expectations of them. Women felt that cooperating with and respecting health workers precipitated better interactions with them, leading to positive experiences. They agreed that obeying instructions, speaking politely and offering truthful responses were helpful for the health workers as they discharge their duties (Table 3, P1, private secondary facility). Asides these, women also viewed the demand for “hospital requirements” and cash deposits on admission as just, given that women are informed about these during the prenatal period (Table 3, P6, public secondary facility). Some mothers opined that offering bribes to health workers in the crowded referral facilities endears them to you, improving birth experience (Table 3, P2, public tertiary facility). Some participants also inferred that it is essential for women to demonstrate confidence in the skills of the health workers. Participants also believed that health workers wanted them to act “normal” without screaming out in pains as this upsets some health workers (Table 3, P9, public primary facility and P3, public tertiary facility).

## Discussion

This study identified the expectations and experiences of mothers who accessed health facility childbirths in Owerri, primarily as the State has continually maintained the highest coverage in Nigeria over the last decade [8,22]. From the findings, it was evident that women had specific expectations of QoC for the type or level of health facility they chose to access care. Women chose each facility following several considerations and trade-offs. Mothers using the public referral facilities prioritised the expertise available and sought care despite assessing the health worker interactions as inadequate. On the other hand, mothers using private facilities prioritised dignified care and, in most cases, the presence of a renowned specialist. Women accessing public primary facilities prioritised cost of care, low patient volume and proximity to their homes. The EoC was not optimal for the participants irrespective of care facilities used. However, the sources of dissatisfaction were anticipated before accessing care. The women demonstrated a good understanding of the expectations of health providers, however, they surmised that most providers do not fully understand or are otherwise constrained in meeting their needs during childbirth.

**Interpretation**: in this study, women appeared to be acquainted with their rights to dignified care and the standard QoC possible during childbirth. However, the negotiations for what is available, acceptable and trade-offs during health facility childbirth appear go on through their prenatal contact with the health facility. While these women witnessed and expressed dissatisfaction at some aspects of care in the prenatal period (e.g. long wait times, verbal abuse), achieving the best possible health outcomes were prioritised and framed the decisions for where they seek care. In appraising the QoC for childbirth, the participants' responses transcended the period of facility confinement but included every contact with the health facility from first antenatal visit till baby immunisation visits. This amplifies the existing body of evidence which suggests that women's experiences through pregnancy, birth and immediate postnatal period are a *"psychological and physical continuum"* [23]. It has been reported that the QoC during childbirth, which encompasses the EoC may be undermining the efforts to increase health facility deliveries [11,24]. Though difficult to define and measure [25], the experience of childbirth has continued to gain attention following the WHO statement in 2014, promoting women's right to respectful and dignified childbirth experiences [26]. Different forms of disrespectful care were reported at all levels and types of care. As per a 2017 systematic review, verbal abuse and lack of privacy appeared to be the most common forms of disrespectful care reported by mothers in Nigeria during childbirth [27]. Many women attribute the higher prevalence of verbal abuse in the public health facilities to the absence of systems to identify cases and hold health workers accountable [28]. With the focus on good outcomes and the foreknowledge of the negative aspects of care, it was not surprising that some participants accepted mistreatment as a necessary price for good outcomes. The role of the mothers in reinforcing the supposed benefits of *"normative"* physical abuse (e.g. slapping the thighs) in ensuring good outcomes were also reported [29,30]. These entrenched beliefs constitute a significant barrier in attaining respectful maternity care. It was clear that despite the inadequacies noted, the major attraction for public referral facility use was the expertise and facilities available [31], while client satisfaction stemming from respectful care is the attraction for private facilities [32]. However, it was also highlighted that the current facility childbirth coverage may be driven by the desire to conform with the social norms. With one or more negative experiences reported from different types and levels of care, there is a case for systematically addressing suboptimal childbirth experiences through periodic health worker training and systems which reinforce patient-centredness in care delivery [31]. The ability to pay for maternal health services also shaped EoC for the participants. Some mothers, due to cost opted to utilise the public facilities though desiring the perceived dignified care feasible in private facilities. Each woman who had to pay out-of-pocket considered the projected cost in choosing a health facility. Interestingly, some of these mothers bypassed the primary care in self-referrals to higher levels of care and saw this as a cost-saving mechanism as developing complications in lower facilities would mean multiple out-of-pocket payments. Judging by the Imo ministry of health survey finding in 2019 which showed a 1.7% health insurance coverage and 69% catastrophic expenditure for households in Imo State [33], this coping strategy is not surprising. The bypass of the primary care in Nigeria continues to pose a strain to the health system as referral facilities are over-burdened, while the primary centres which are the supposed care entry points are underutilised [34]. With the poor health insurance coverage, these financial constraints have grave implications for maternal and newborn health outcomes [35]. While mothers who had health insurance coverage in public facilities felt it afforded them uninterrupted care, their counterparts in private facilities felt that having an insurance premium automatically reduced the affection and quality of services from health workers. This, however, varies with findings from a study conducted in Nigeria, which reported better access to benefits packages and QoC for insured clients in private facilities [36]. All participants felt that they had roles in engendering positive childbirth experiences, especially in relating with the health care providers. They tried to be cordial, respectful and truthful to the health workers, and they felt this improved rapport with them. Some mothers also felt compelled to subjugate their feelings of pain, *"be silent"* and act *"normal"* through labour and childbirth to please the health providers [37]. The women generally felt that despite understanding and trying to meet health provider expectations, their own needs were neither fully understood nor met by health care providers.

**Strengths and limitations**: this is the first study conducted in the region with the highest health facility childbirths in Nigeria and compares the EoC for purposively sampled mothers across facility types and cadres, contributing knowledge to an understudied area in literature. Also, participants delivered at least eight weeks before the study, reducing the chances for a “halo effect”, which is a wrong assessment of care received in the immediate postpartum attributed to the joy of a successful birth [38]. To enhance the usefulness of this study for other researchers, we employed reflexivity, triangulated via data sources, (compared accounts across similar facilities and also across caregivers and users in the sample population), used iterative questioning, reflective field notes, peer debriefing sessions, member checks to attain trustworthy findings. Limitations include the scope as participants were recruited from only three of 27 Local Government Areas (LGAs) in the State. However, the diversity of participants and facility types suggest that findings may not vary in other LGAs. Again, the researchers' position as health workers interviewing participants for experiences of care created room for potential social desirability bias [16]. However, the researchers employed direct and indirect (referencing a third-party) questioning as necessary to elicit honest answers.

## Conclusion

Every woman who overcomes the known access barriers and arrives at a health facility in labour deserves a positive childbirth experience. To achieve this, mothers' perceptions of different providers need to be heard. Health workers must remember that holistic care delivery can only be achieved when they render skilful, timely, and yet empathetic care which will help women feel more in control and as partners in their care. Going forward, strategies which leverage the strengths of the private and public providers must be engaged and upscaled if we must reach the maternal mortality reduction target of the Sustainable Development Goal 3.

### What is known about this topic

- Nigeria has poor facility childbirth coverage (39.4%), and Imo State has maintained the highest coverage (95.4%) in the last decade;
- When mothers have negative experience (such as physical abuse, etc.) during childbirth, it may hinder future health facility utilisation.

### What this study adds

- The experience of care is staggered around health provider types and level of health facilities. However, one or more forms of negative experiences were reported in all types and levels of care.
- Though women desired providers who are experts and rendered respectful, responsive care, they are often forced to make trade-offs between expertise and respectful maternity care.
